# Spatial Variation in Turf Surface Properties of Polo Pitches: A Case Study of Different Handicaps of Argentina

**DOI:** 10.3390/ani16040685

**Published:** 2026-02-22

**Authors:** María Alejandra Blanco, Michael L. Peterson, Pablo Ariel Cipriotti, Fernando Apecechea

**Affiliations:** 1 Facultad de Ingeniería y Ciencias Agropecuarias, Pontificia Universidad Católica Argentina, Buenos Aires B1300, Argentina; 2 Biosystems and Agricultural Engineering, University of Kentucky and Racing Surfaces Testing Laboratory, 907 National Ave, Lexington, KY 40502, USA; mick.peterson@uky.edu; 3 Facultad de Agronomía, Universidad de Buenos Aires, Av San Martín 4453, Buenos Aires B1417, Argentina; cipriott@agro.uba.ar; 4 Escuela Superior de Ingeniería, Informática y Ciencias Agroalimentarias, Universidad de Morón, Gral. Machado 854, Morón, Buenos Aires B1708, Argentina; fernandoapecechea@hotmail.com

**Keywords:** polo, natural turf, handicap base level, mechanical response, spatial analysis, equine safety, welfare

## Abstract

Polo is a high-speed equestrian sport that imposes mechanical demands on horses and turf, yet limited research has examined the functional behavior of polo playing surfaces. This study characterizes the spatial variability of mechanical surface properties across turf polo pitches representing high-, medium-, and low-handicap categories. Three fields were assessed using lightweight field-based instruments, including the Impact Test Device (ITD), Rotational Peak Shear (RPS) tester, Going Stick© for penetration (GSP) and shear (GSS), and a TDR probe for volumetric moisture content (VMC%). A total of 210–223 grid-based sampling points per pitch were analyzed to evaluate mechanical responses under vertical and horizontal loading conditions. Significant differences among pitches were observed, with ITD and VMC emerging as the indicators of surface behaviour. Spatial analysis revealed heterogeneous within-pitch patterns, expressed as directional gradients and localized variability. Linear discriminant analysis demonstrated that the combined measurements could differentiate pitches associated with different handicap levels with high classification accuracy (0.88). Although the applied instruments do not replicate full equine biomechanical loading, they proved effective in detecting spatial variability in surface uniformity, a functional property relevant to performance and equine welfare. These findings support integration of spatially explicit surface assessments into routine turf management practices.

## Simple Summary

This study shows that spatial variability in polo fields can be evaluated using low-cost, portable tools, providing clubs and governing bodies with practical methods for routine surface monitoring. Identifying volumetric moisture content (VMC%) and impact firmness (ITD) as key indicators supports the development of objective, evidence-based criteria for field maintenance and assessment. Detecting variability within pitches, particularly on natural soil surfaces, allows more efficient allocation of resources to improve surface consistency, playability, and equine welfare. The ability to classify fields by handicap level with high accuracy (85–90%) offers a data-driven framework for benchmarking and performance auditing. Overall, the use of lightweight tools facilitates regular monitoring and supports a shift toward empirical, field-based evaluation practices, contributing to improved management decisions and the long-term quality and sustainability of polo playing surfaces.

## 1. Introduction

Polo is a high-speed equestrian sport played on a grass pitch up to 180 m wide and 250 m long. Each team consists of four riders and their horses, with both men and women competing together [1]. A match lasts approximately two hours and is divided into four to six seven-minute chukkers. Players change horses between or during chukkers, typically bringing one horse per chukker and one to three additional horses as substitutes. Polo ponies must execute high-intensity movements and withstand substantial impacts, demands that are distinctive to the sport. Despite its global appeal and the high value placed on the game, literature on equestrian polo remains scarce [2], particularly considering its historical significance and the substantial investment from the polo community [3]. The sport is governed by an international federation and, like other high-level disciplines, is subject to the rules and regulations of the World Anti-Doping Agency (WADA) [4]. Safety is a major concern in polo, and this is reflected in the regulations, which prioritize not only the welfare of the players but also that of the ponies [2]. Additionally, polo regulations require players to interact with a relatively large number of ponies per game compared to other equestrian disciplines [5]. As polo is played on the largest pitch in professional sports (145 m × 275 m), horses can achieve high speeds and cover significant distances [6]. According to Hurlingham Polo Association (2019) [7], polo players are assigned a handicap (−2 to 10 goals), which serves as a quantitative measure of playing ability, incorporating horsemanship, individual and team playing skills, and the quality of ponies used. The level of Polo play is determined by the cumulative handicap of all four team members (e.g., a 10-goal team), allowing for varied combinations of player skill levels [6]. Polo pitches are found worldwide and are primarily built on sandy-loam soils or natural soils with sand added through topdressing. Maintenance is a key factor in ensuring quality, with topdressing and aeration being two of the most important practices for providing adequate support [8,9,10,11,12]. In general, polo pitch quality is classified by handicap team level.

Polo tournaments are classified according to the combined team handicap. Low-handicap tournaments include teams with handicaps from 0 to 10 goals, medium-handicap tournaments range from 11 to 15 goals, and high-handicap tournaments involve teams with 16 goals or more. Within the high-handicap category, tournaments contested at 26– 40 goals, such as the Argentine Open Championship, are defined as the elite level of the sport based on the official handicap system [4,7,13].

An analysis, of spatiotemporal characteristics across 0–24 goal polo, reported that high-intensity activity is a consistent feature of match play, with distance covered, average speed, and high-speed distance increasing as cumulative player handicap increases [6]. Differences in those variables were more pronounced between lower handicap levels (0– 6–10 goals) and higher levels of play, while values at the intermediate level (16 goals) showed partial overlap. These findings demonstrate that the physical demands experienced by polo ponies differ across handicap levels and are relevant to the definition of pitch surface qualities.

Polo ponies experience substantial physiological stress during both match play and training, as demonstrated by spatiotemporal workload analyses across competitive levels [6,14]. Musculoskeletal injuries in polo are commonly associated with repetitive strain, affecting the superficial digital flexor tendon as well as the collateral ligaments of the distal interphalangeal joint [15]. Poor pitch condition has been identified as a major injury risk factor, accounting for 58.6% of reported cases [15,16], while hard or uneven surfaces have been shown to increase tendon and joint injury risk across equestrian sports [17,18].

Functional properties have been developed to characterize equine sport surfaces, particularly in horse racing and disciplines such as show jumping and dressage [19]. Although the concept of functional properties is relatively recent in equestrian sports and horse racing, it has not yet been formally adapted to polo. Surface conditions have been shown to significantly influence injury risk in both horses [20,21] and jockeys [22]. In horse racing, a clear association between track surface conditions—commonly referred to as the *going*—and an increased risk of fractures on turf tracks has been reported in New Zealand [23]. As a result, quantitative surface assessment has been increasingly adopted in equestrian sports, including by the International Equestrian Federation (FEI) [24] and North American horse racing authorities [25]. These initiatives define a set of functional surface properties, including firmness, cushioning, rebound, grip, uniformity, and consistency [20,26,27], which are quantitatively assessed using the Orono Biomechanical Surface Tester (OBST), considered the gold standard for FEI applications and horseracing in the United States [14]. The use of turfgrass as an equine sports surface adds further complexity to horse–surface interactions, as it represents a living and dynamic system whose mechanical properties vary over time in response to management practices, environmental conditions, and use.

Despite the demanding nature of polo and the well-established influence of surface quality on safety in equestrian sports, there is limited research on turf surfaces for polo. Measuring relevant surface parameters with instruments that replicate the loads and speeds of the horse is ideal [21,22,28]. However, the high costs and logistical challenges associated with instruments of sufficient size and mass to replicate equine loading limit their adoption beyond the elite levels of the sport. Smaller, more accessible tools could facilitate surface quality control across a broader range of contexts, promoting improved maintenance and design practices. In other equestrian disciplines, such as horse racing, research has examined the effects of surface type and condition on injury incidence and performance [29,30,31,32]. While quantitative surface assessments exist, track ratings often rely on subjective evaluations by officials, leading to inconsistencies across jurisdictions [33]. In contrast, New Zealand employs strictly quantitative methods, which have proven useful for both performance evaluation [34,35] and risk assessment [23,36]. In disciplines like show jumping and dressage, the relationship between surface conditions and injuries is less well documented, yet it is widely acknowledged that a better understanding of equestrian turf surfaces is essential for ensuring horse and rider safety [19,20,27].

Polo pitches are commonly established on native soil substrates, which inherently exhibit spatial heterogeneity. As reported by Straw et al. [37,38,39], natural turfgrass sports surfaces display substantial within-field variability driven by climatic conditions, construction practices, maintenance protocols, and the spatial distribution of mechanical loads associated with repeated use. In polo, such site-specific variability can reduce surface uniformity and predictability, necessitating frequent or abrupt adjustments by both horse and rider. These adaptive responses increase mechanical loading on the musculoskeletal system and may elevate the risk of surface-related injury [40,41]. Accordingly, quantifying and managing intra-field variability is essential for maintaining functional surface performance and supporting the safety and performance of both equine and human athletes.

Research on equestrian surfaces has primarily focused on disciplines such as thoroughbred racing and show jumping, where the adoption of standardized measurement methods for assessing surface functionality is increasing, particularly in U.S. horseracing and FEI CSI4* and CSI5*level competitions [19].

This study aimed to (i) quantify and compare functional surface properties across three polo pitches representing different levels of play defined by cumulative team handicap, (ii) evaluate the discriminative capacity of field-based measurement instruments to detect surface-level differences relevant to handicap-based competition categories, and (iii) characterize within-field spatial heterogeneity of surface properties in relation to surface functionality and equine safety. The functional properties defined by the OBST are linked to underlying mechanical surface properties that can be estimated using small and lightweight field-based devices [42,43,44]; although the dynamic loading conditions of these instruments are not equivalent to those applied by the OBST, some devices can still describe mechanical properties that are useful for characterizing surface condition.

This study represents the first known effort to systematically apply a suite of portable, low-cost instruments to evaluate the spatial variation in functional properties of turf polo playing surfaces under real field conditions.

## 2. Materials and Methods

### 2.1. Site Description and Pitch Classification

In May 2019, three polo pitches—classified as high, medium, and low handicap—were selected from a total of twelve located at a major polo facility in the Buenos Aires region. The soil at the study site is classified, according to Soil Conservation Services (1994), as a Vertic Argiudoll, fine-slope phase, illitic, thermic. The terrain is characterized by a transverse slope descending from west to east, and a longitudinal slope descending from south to north.

The landscape consists of elongated hills and gently undulating plains. The natural slopes across the site, where the polo pitches are located, average approximately 4%, with a transverse gradient oriented from southwest to northeast (SW–NE) and a longitudinal gradient from northwest to southeast (NW–SE). These grounds were constructed approximately ten to twenty years prior on the native silty clay loam soils and are maintained following the AAP’s regular recommendation management practices. The region has a temperate humid climate, with mild to cool winters, warm to hot summers, and a mean annual rainfall of approximately 950 mm. The playing season typically extends from spring through early summer. The turfgrass used on all pitches is ‘Tifway’ Bermudagrass (*Cynodon dactylon* (L.) Pers. × *C. transvaalensis* Burtt Davy). All surface assessments were conducted prior to the winter dormancy period. Due to the extensive area of each pitch, testing on each surface was completed over the course of one to two days. The polo pitches were irrigated using a two big-gun sprinkler irrigation system, with irrigation suspended the day before testing. Ten soil subsamples were collected with a soil sampler of 0.20 m from each polo pitch following a systematic diagonal transect sampling design, with sampling points spaced at 50 m intervals and subsamples taken alternately 1 m to either side of the transect and analyzed for fertility (Appendix A). Monthly precipitation and air temperature data (mean minimum, mean, and mean maximum), measured in a meteorological shelter at 1.50 m height (Appendix A).

Pitch categories (high, medium, and low handicap) were originally established by the pitch managers at the time the pitches became operational, based on combined assessments of soil and turf quality.

**Figure 1:**
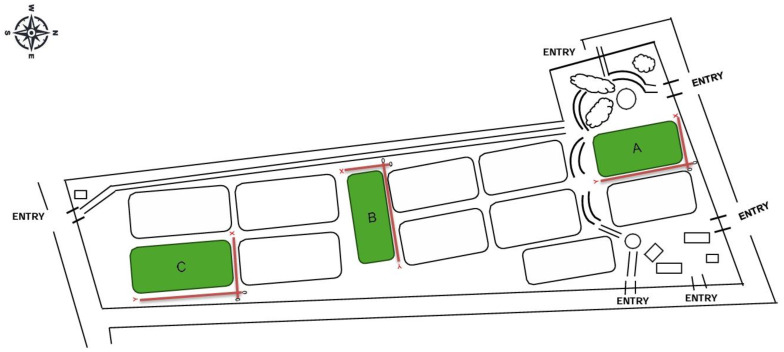
Layout of the polo pitches at a major facility ground. The facility comprises twelve pitches organized in a grid layout with multiple access points. The pitches highlighted in green (A, B, and C) represent those selected for surface evaluation in this study. Pitch positions are referenced according to their orientation and proximity to main entries.

Polo pitch A and C measured 150 m × 280 m (42,000 m^2^) and polo pitch B measured 135 m × 300 m (40,500 m^2^). Polo pitch A was representative of high-handicap level surfaces, polo pitch B corresponded to medium handicap, and polo pitch C to low handicap. The x and y axes are defined in Figure 1 to indicate the starting point of the test.

### 2.2. Surface Grid Design and Data Collection

The playing surfaces were subdivided into a 10 m × 24 m grid, resulting in a total of 223 plots on polo pitches A and C, and 210 plots on pitch B. Each plot represented approximately 0.47% of the total pitch area. Measurements were taken from morning to afternoon. To evaluate both vertical and horizontal surface responses, either multiple instruments or multi-axis devices were required. The Going Stick© (Turftrax Ltd., Cambridgeshire, UK) is a device widely used on turf surfaces for Thoroughbred racing, especially in the United Kingdom, to measure penetration and shear resistance. Portable tools based on ASTM standards were also employed to characterize mechanical behavior. These included: the Impact Test Device (ITD) [45]. The Rotational Peak Shear (RPS) tester [46], to evaluate rotational shear strength; a Time–Domain Reflectometry (TDR) sensor [47], for determining volumetric moisture content. One measurement with each tool was conducted in each plot. For each test plot, one measurement was taken at equidistant locations within the inner area of the plot, avoiding edges to reduce boundary effects. The Going Stick provided measurements of penetration resistance (GSP) and longitudinal shear (GSS), which were converted to newtons (N) and Newtonmeters (Nm), respectively, using the calibration equations described in Blanco et al. (2021) [42]. The ITD equipped with a Slam Stick^®^ (200 G-1 GB) to assess surface deceleration, was dropped five times per plot, and the highest value among the four valid readings was retained [45].

#### 2.2.1. The Going Stick©

**Figure 2:**
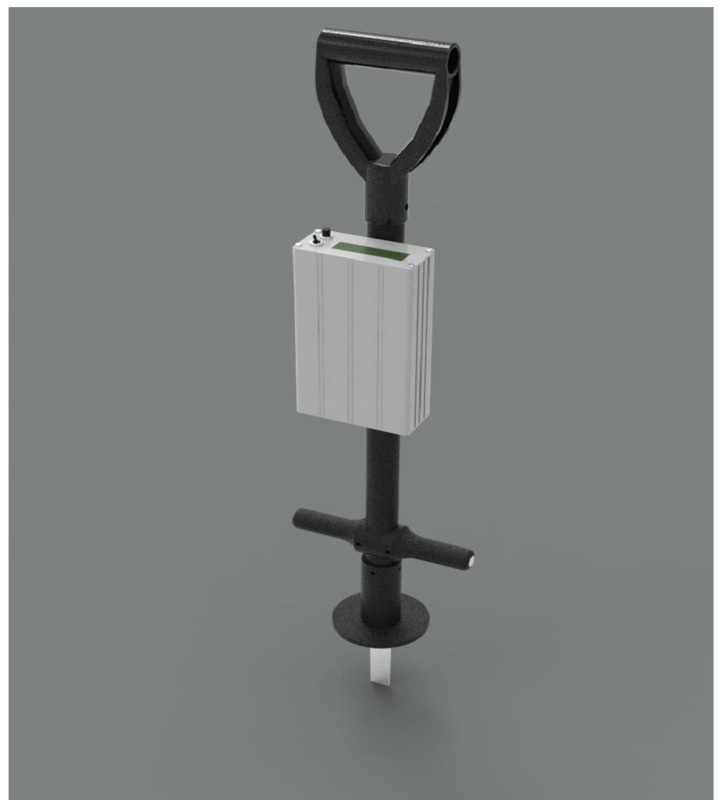
The Going Stick©, a field instrument designed to quantify the penetration resistance and the longitudinal shear for turf tracks by inserting a probe into the surface and rotating it through a fixed angle of 45 degrees.

The Going Stick© is a portable field instrument developed to quantify the mechanical properties of turf surfaces, specifically penetration resistance (GSP) and shear strength (GSS) (Figure 2). Penetration is defined as the peak force required to insert the 100 mm probe vertically into the surface, while shear strength (longitudinal) is derived from the maximum torque needed to rotate the probe 45°, both measured via integrated strain gauges positioned 128 mm from the probe tip [48,49]. Following calibration with known loads using a standardized fixture [42], these outputs are expressed in Newtons (N) and Newton-meters (Nm). A composite metric, referred to as the going index, is calculated from the force and torque measurements and is commonly used in Thoroughbred racing in the United Kingdom to characterize surface conditions. Although the tapered geometry of the probe and the lack of horizontal constraint introduce complexity in interpreting the applied stresses, simplified assumptions—such as a fixed rotational axis at the turf interface—allow for consistent estimations suitable for comparative analysis. In this study, all measurements were performed using software version 2.30, which stores peak (non-averaged) values for both parameters. The device was operated in flat mode throughout data collection.

#### 2.2.2. The Impact Test Device (ITD)

**Figure 3:**
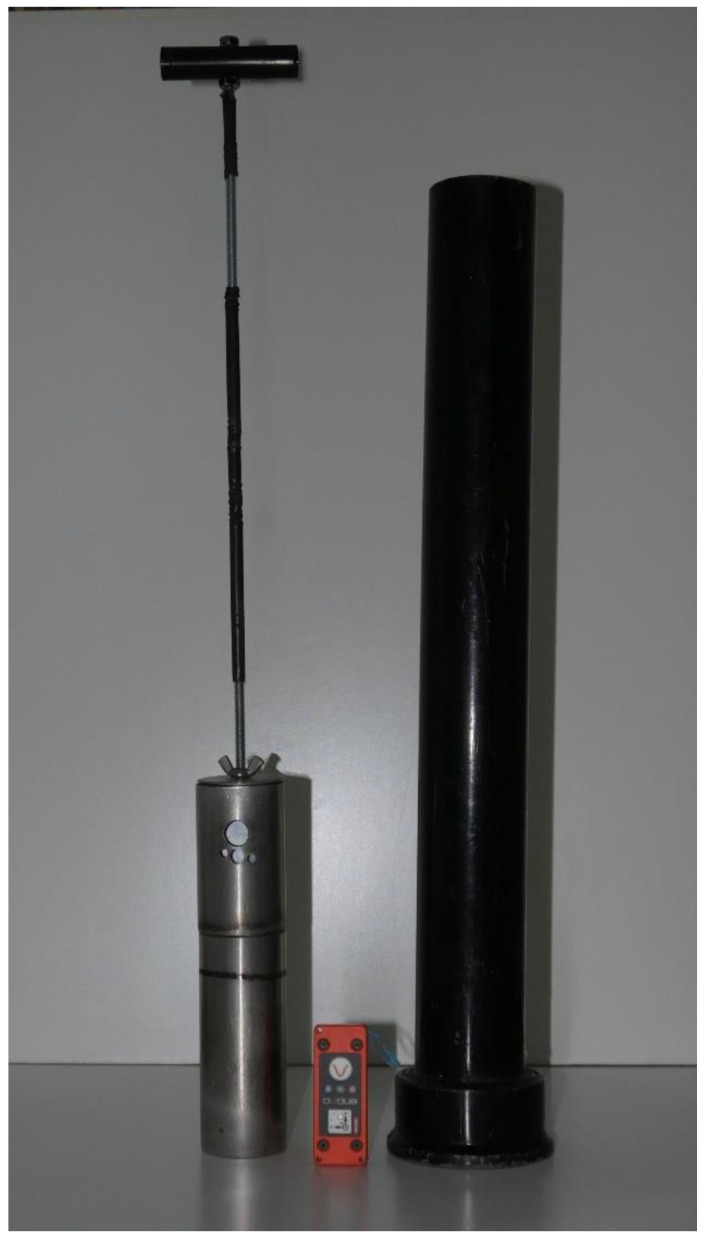
Impact Test Device (ITD) designed according to ASTM D5874-16. The ITD comprises a 2.25 kg mass that is released from a height of 0.45 m within a rigid vertical guide tube. A high-resolution accelerometer and data logger (Slam Stick^®^ 200 G-1 GB, Mide Technology, USA) is integrated within the device to record acceleration profiles during impact events.

Surface hardness and compaction resistance were evaluated using a custom-built Impact Test Device (ITD), designed in accordance with ASTM D5874-16 [45] (Figure 3). The ITD consists of a 2.25 kg mass dropped from a height of 0.45 m within a rigid vertical guide tube. It quantifies surface response by recording the vertical deformation at each impact, yielding the Impact Test Device Coefficient (ITDC), which reflects the surface’s ability to absorb and dissipate energy under dynamic loading. The device is internally equipped with a Slam Stick^®^ 200 G-1 GB (Mide Technology Corporation, Woburn, MA 01801, USA) a high-resolution accelerometer and data logger, previously calibrated, which captures acceleration profiles during impact events. This addition enables detailed analysis of impact dynamics and surface stiffness characteristics. All the equipment was previously calibrated on concrete. Each measure consisted of four successive drops conducted at a single location. The vertical acceleration was recorded for each drop using the Impact Test Device (ITD), and the maximum acceleration value among the four drops was recorded for analysis to represent the impact response of the surface, in accordance with ASTM F5874-16 [45].

#### 2.2.3. The Rotational Peak Shear

**Figure 4:**
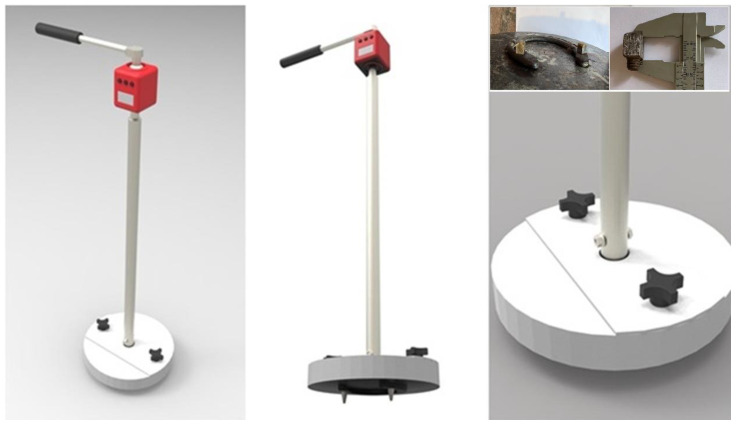
Rotational traction testing device (RPS), adapted from equipment originally developed for sports fields, as described in ASTM F2333-04. The apparatus measures the resistance to rotational movement on natural or synthetic surfaces by applying a controlled torque. Polo cleats detail in right superior corner.

Rotational shear strength was assessed using a custom-built device based on a modification of ASTM F2333-04 [46] (Figure 4), in which the standard cleats were replaced with a steel horseshoe. To pre-set the testing disk onto the surface, a 30 kg mass was dropped from a height of 0.30 m along a vertical shaft onto a secondary plate. This plate had a size 3 steel horseshoe affixed to its underside, which featured two standard polo pony cleats, which are square in shape with dimensions of 0.010 m × 0.011 m along their abaxial face. The rotational peak shear (RPS) load was measured using a digital torque wrench (Model ARM602-4, ACDelco, Taiwan, China) with a measurement range of 4–200 Nm and a precision of 0.08 Nm. To determine the failure load, the torque wrench was rotated manually until the maximum torque was reached, while ensuring that the plate remained flat against the surface throughout the test. One replicate was performed for each plot. To minimize operator-related variability, all measurements were conducted by the same individual, following established best practices for mechanical surface testing.

#### 2.2.4. Moisture Measurement with Time Domain Reflectometry (TDR)

Volumetric moisture content (VMC, expressed as a percentage) was measured using time domain reflectometry (TDR), as described in ASTM D6780-19 [47] a widely accepted method for assessing soil water content and a key variable influencing surface consistency. Measurements were performed using a TDR moisture probe (Field Scout TDR-100, Spectrum Technologies, Aurora, IL, USA) equipped with two stainless steel rods, each 0.07 m in length. The probe was inserted vertically into the surface to ensure full contact of the rods with the substrate, allowing for accurate and repeatable readings of VMC.

### 2.3. Data Processing and Statistical Evaluation

The data collected from all instruments were analyzed at three hierarchical levels, each corresponding to a specific study objective.

At the between-field level, analyses were conducted to quantify and compare mean functional surface properties among the three polo pitches. Descriptive statistics and visual inspections (box plots) were first performed for each polo pitch. Data normality was assessed using both the Kolmogorov–Smirnov and Shapiro–Wilk tests, while homogeneity of variances was evaluated with Levene’s test. For variables meeting normality and homoscedasticity assumptions (RPS), one-way ANOVA to detect differences on means was applied, followed by Tukey’s post hoc test when significant differences were detected. For non-Gaussian variables (GSP, GSS, ITD, and VMC), the Kruskal–Wallis test was used to detect differences on medians. Additionally, pairwise two-sample Kolmogorov–Smirnov tests were applied to compare the empirical distributions of each variable among polo pitches (Pitch A vs. Pitch B, Pitch A vs. Pitch C, and Pitch B vs. Pitch C). This approach enabled an integral assessment of differences based on max distance (D stat), not only in central tendency but also in the shape and spread of the distributions across playing surfaces.

To assess the ability of field-based instruments to discriminate among polo pitches representing different handicap levels, a multivariate analysis using Linear Discriminant Analysis (LDA) was performed. Prior to LDA application, variogram analysis was conducted for each response variable to evaluate the presence and scale of spatial autocorrelation, thereby confirming that samples could be treated as independent observations for subsequent multivariate analysis [50]

At the within-field level, surface regression analysis based on full quadratic models were applied to describe internal spatial heterogeneity within each field, allowing identification of localized variability relevant to surface functionality and equine safety. All statistical analyses were performed using Statistica (Statsoft 9.1), Navure 2.6.1 (Universidad Nacional de Córdoba, Argentina) and R (R Core Team, 4.4.1) and rsm package 2.10.6 [51].

## 3. Results

### 3.1. Comparative Analysis of Surface Mechanical and Moisture Parameters Across Three Polo Pitches

All evaluations from the five parameters respectively capture penetration resistance, shear strength, and impact-related surface properties. The results are described through boxplots for each tool, offering a clear visualization of the median, variability, and the distribution of atypical values, including outliers and extremes.

#### 3.1.1. The Going Stick

**Figure 5:**
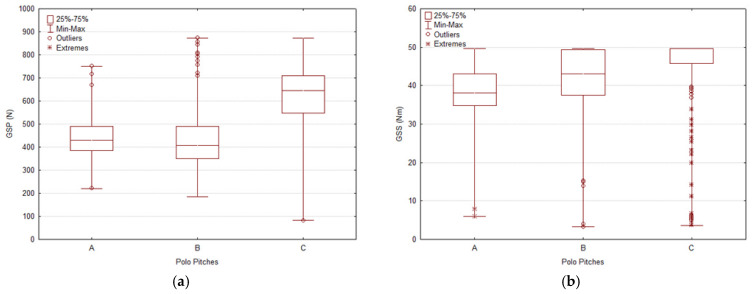
Boxplots of mechanical surface variables measured across three polo pitches (A, B, C). (**a**) shows the distribution of penetration resistance (GSP) and (**b**) and longitudinal shear resistance (GSS) (**b**). Boxes represent interquartile ranges (25th–75th percentile), with medians indicated by horizontal lines. Whiskers denote minimum and maximum values excluding outliers, which are displayed as circles (mild) and asterisks (extreme). The results illustrate inter-pitch differences in both central tendency and variability for GSP and GSS.

Penetration from the Going Stick (GSP) (Figure 5a) shows that Pitch C exhibited the highest median GSP values and the widest overall range, indicating a firmer but more variable surface. The abundance of outliers in Pitch C indicates greater within-field variability in penetration resistance. In contrast, Pitches A and B presented lower median values and narrower interquartile ranges (IQR) than Pitch C (A = 427.84 N; B = 407.47 N; C = 646.13 N; H = 232.73, *p* < 0.0001), indicating reduced variability and a softer, more consistent surface profile.

Shear from the Going Stick (GSS) (Figure 5b) showed that Pitch C exhibited values tightly clustered around the median (A = 37.93 Nm; B = 43.07 Nm; C = 49.70 Nm; H = 136.10, *p* < 0.0001), while also displaying a skewed distribution characterized by a long lower tail with several extreme low-end values, indicating localized areas with very low shear resistance. Pitch B exhibited the broadest overall range and a high number of outliers, suggesting uneven longitudinal traction conditions, whereas Pitch A showed intermediate values with lower variability, consistent with a more predictable shear profile.

#### 3.1.2. The Impact Test Device (ITD)

**Figure 6:**
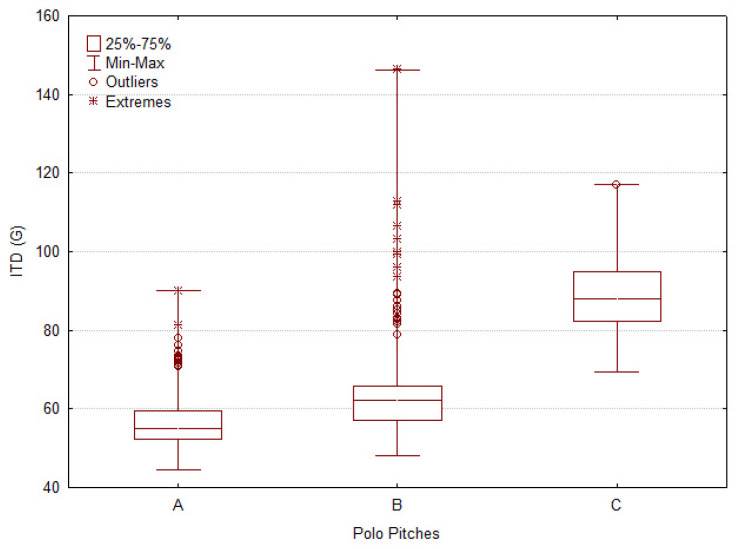
Boxplots of mechanical surface variables measured across three polo pitches (A, B, C). (%). Figure shows the distribution of Impact Test Device Coefficient (ITDC). Boxes represent interquartile ranges (25th–75th percentile), with medians indicated by horizontal lines. Whiskers denote minimum and maximum values excluding outliers, which are displayed as circles (mild) and asterisks (extreme). The results illustrate inter-pitch differences in both central tendency and variability for ITD.

Results for ITD showed that Pitch A (Figure 6) exhibited the lowest median ITDC values and the greatest consistency, as reflected by a narrow interquartile range (A = 54.97 G; B = 62.11 G; C = 88.99 G; H = 421.79, *p* < 0.0001), indicating a firm surface with uniform shock absorption characteristics. Pitch B showed the greatest overall range and highest number of outliers, implying highly variable impact absorption capacity. In contrast, Pitch C (Figure 6) presented the highest median ITDC values together with a wider interquartile range, indicating greater variability in the central impact response despite fewer extreme values.

#### 3.1.3. The Rotational Peak Shear (RPS)

**Figure 7:**
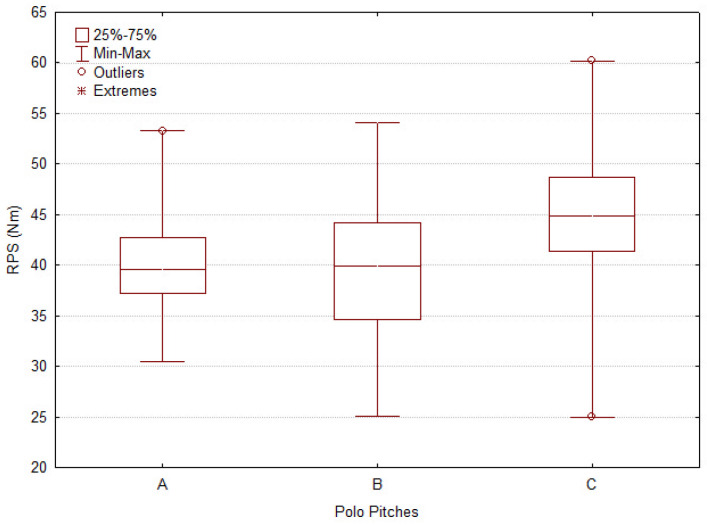
Boxplot of mechanical surface variables measured across three polo pitches (A, B, C). (%). The Graph show the distribution of Rotational Peak Shear (RPS). Boxes represent interquartile ranges (25th–75th percentile), with medians indicated by horizontal lines. Whiskers denote minimum and maximum values excluding outliers, which are displayed as circles (mild) and asterisks (extreme).

Pitches A and B (Figure 7) exhibited intermediate median RPS values (A = 39.90 Nm; B = 39.60 Nm), whereas Pitch C showed a higher median RPS value (44.85 Nm; F = 64.57, *p* < 0.0001) together with a broader spread. The wider interquartile range observed for Pitch C indicates greater variability in rotational shear resistance, reflecting stronger resistance to rotational movement but reduced surface evenness compared with Pitches A and B.

In contrast, Pitch A displayed the lowest median RPS values and the narrowest interquartile range, indicating lower shear strength but more uniform rotational behavior.

#### 3.1.4. Volumetric Moisture Content (VMC%)

**Figure 8:**
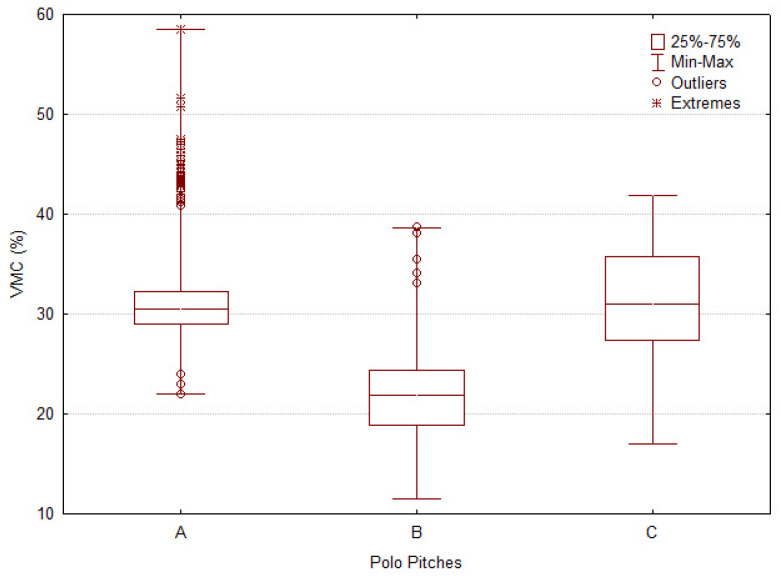
Boxplot of mechanical surface variables measured across three polo pitches (A, B, C). Figure shows the distribution of Volumetric Moisture Content (VMC). Boxes represent interquartile ranges (25th–75th percentile), with medians indicated by horizontal lines. Whiskers denote minimum and maximum values excluding outliers, which are displayed as circles (mild) and asterisks (extreme).

Pitch A (Figure 8) exhibited high median moisture content with a broader overall range and a high density of outliers, indicating substantial within-field variability. Pitch B showed the lowest median VMC and the narrowest distribution, reflecting drier and more uniform moisture conditions. Pitch C also displayed a high median VMC; although comparable to Pitch A, its distribution was narrower and outliers were less frequent, indicating lower heterogeneity (A = 30.50%; B = 21.92%; C = 31.00%; H = 285.81, *p* < 0.0001). These results indicate that Pitch C provided the most uniform moisture profile, whereas Pitch A exhibited considerable internal variation that may require improved irrigation or drainage strategies. Pitch C therefore appears to balance surface uniformity with natural moisture variability.

### 3.2. Application of Linear Discriminant Analysis to Classify Polo Fields Based on Mechanical Surface Properties

**Figure 9:**
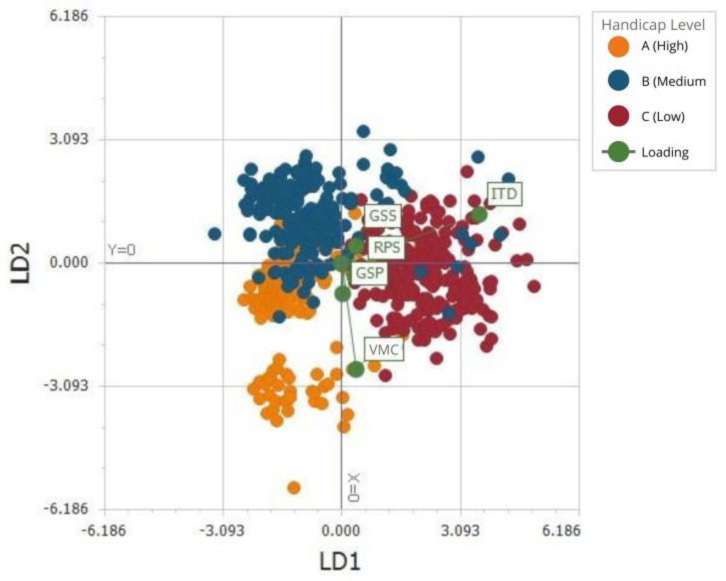
Linear discriminant analysis (LDA) of surface condition data from three handicap levels pitches (Pitches A, B, and C), based on variables measured with portable assessment tools (green markers). Each point represents a sampling location, color-coded by pitch. The first two linear discriminants (LD1 and LD2) illustrate the separation among pitches and the multivariate contribution of each tool. The observed clustering indicates that the tools captured sufficient variation to differentiate pitch conditions, with Pitches A and C showing greater internal dispersion compared to Pitch B. These patterns suggest differences in surface uniformity and highlight the discriminative power of the selected variables.

The evaluated tools effectively differentiated among the three handicap levels analyzed, achieving global accuracy of 0.88 with a 95% confidence interval of 0.85–0.90, obtained by resampling. ITDC exhibits greater discriminative power than GSP and CHV, as reflected by its stronger loadings along LD1, while GSP, GSS, RPS and VMC contribute more weakly and primarily describe within-group variability. The first linear discriminant function (LD1; Figure 9) effectively distinguished between low (C) and intermediate-handicap pitches (B) and was primarily associated with ITD measurements, with the highest values observed in the lowest-handicap pitch (C). In contrast, greater within-field variability in the highest-handicap pitch (A) was captured along the second linear discriminant function (LD2; Figure 9), mainly associated with VMC (%), which showed the strongest negative correlation with this axis.

### 3.3. Spatial Characterization of Mechanical Surface Properties Within Polo Pitches

Two-dimensional response surfaces were generated to characterize within-field spatial heterogeneity of the measured mechanical variables across the three polo pitches classified by handicap level (Appendix C).

**Figure 10:**
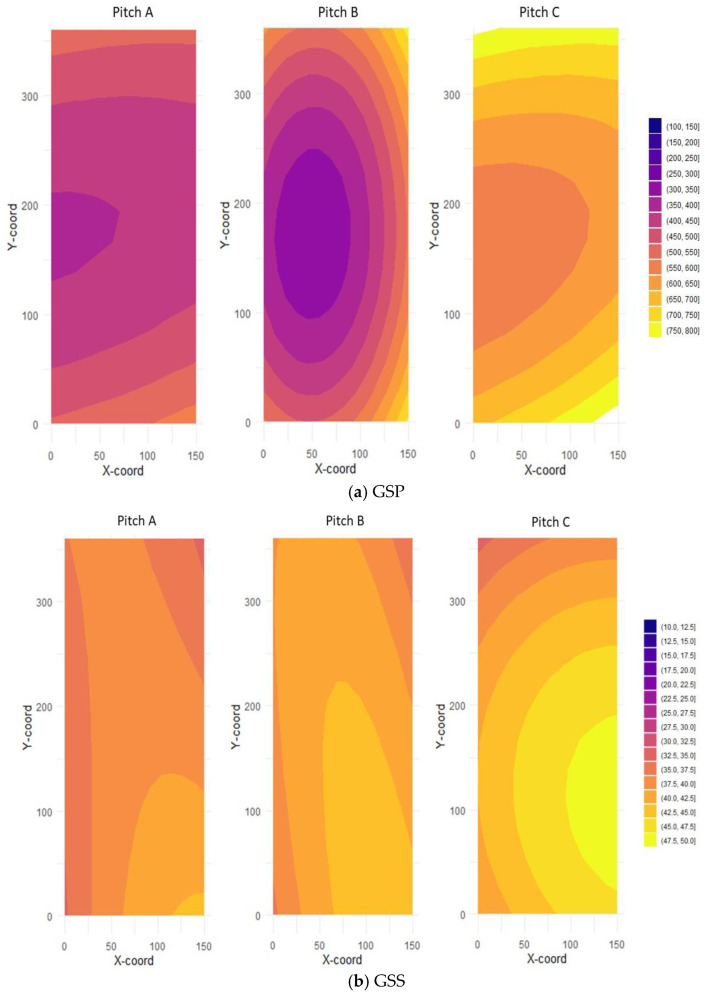

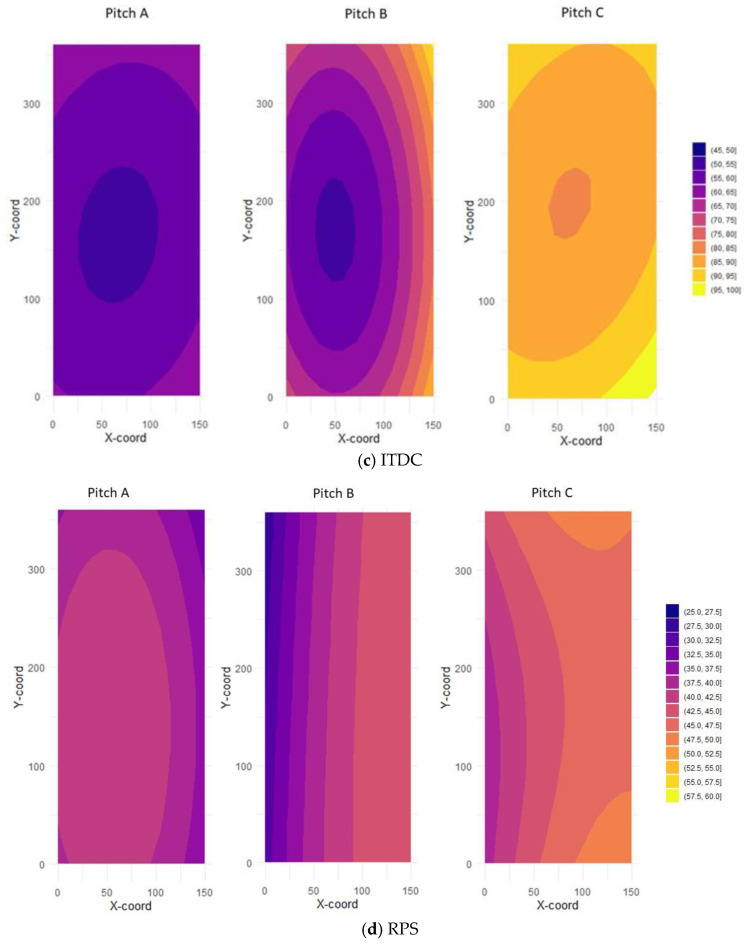

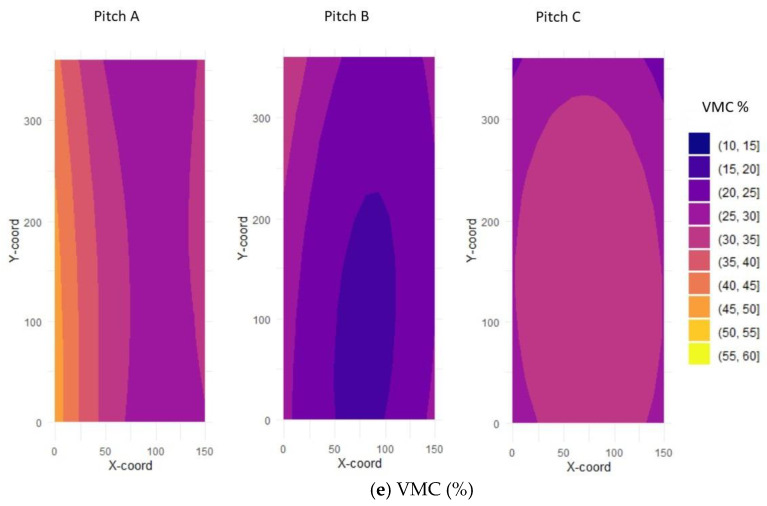
Spatial distribution of Penetration (GSP), and longitudinal shear (GSS), Impact Test Device coefficient (ITDC), Rotational Peak Shear (RPS) and Volumetric Moisture Content (VMC %), across three polo pitches (Pitches A, B, and C). The 2D surface plots illustrate the spatial variability of each parameter. (**a**) For GSP, harder zones (>750 N) are shown in yellow, to softer (>250) zones appear in purple indicating moderate-to-strong spatial dependence of mechanical and physical surface properties mainly within Pitch B (R^2^a = NS; R^2^b = 0.36; R^2^c = 0.12); (**b**) For GSS, from low (<25 Nm) are shown in purple to high (>45 Nm) appear in yellow longitudinal shear (R^2^a = NS; R^2^b = NS; R^2^c = NS); (**c**) For ITDC, softer zones 45–50 G are shown in purple while harder zones (>95G) appear in yellow, indicating moderate-to-strong spatial dependence of mechanical surface properties within Pitch B (R^2^a = NS; R^2^b = 0.49; R^2^c = 0.10); (**d**) For RPS, from low (>25 Nm) are shown in yellow, to moderate (>45 Nm) zones appear in purple indicating moderate-to-strong spatial dependence of mechanical surface properties mainly within Pitch B (R^2^a = 0.24; R^2^b = 0.45; R^2^c = 0.17). (**e**) For VMC, wetter zones (>50%) are shown in yellow, while drier areas (<18%) appear in purple. The surface model explains a substantial proportion of the observed spatial variation (R^2^a= 0.62; R^2^b = 0.33; R^2^c = 0.13), indicating moderate-to-strong spatial dependence of mechanical surface properties mainly within Pitch A.

The response surface analysis revealed that most parameters recorded by the instruments exhibited spatially structured patterns within the handicap-level polo pitches, expressed as gradual spatial gradients and localized patches rather than random variation. For volumetric moisture content (VMC), regression coefficients indicated a pronounced spatial gradient in Pitch A (R^2^ = 0.65), primarily aligned with the *Y*-axis (Figure 10a). In contrast, spatial structure was less pronounced in Pitch B (R^2^ = 0.33) a more localized, patch-like spatial pattern and Pitch C (R^2^ = 0.13) displayed a relatively homogeneous moisture distribution with no dominant directional gradient.

Despite variations in moisture content, no strong or consistent spatial gradients were observed for ITDC or GSP measurements across the pitches, as indicated by the absence of significant large-scale spatial correlations.

At the individual pitch level, Pitch B exhibited moderate coefficients of association for ITDC (R^2^ = 0.49), rotational shear (RPS; R^2^ = 0.45), and penetration resistance (GSP; R^2^ = 0.36), with patterns oriented toward the longitudinal side of the field (Figure 10b–d). These associations indicate weak but detectable spatial structure rather than pronounced gradients.

For GSS measurements, variability did not exhibit a clear spatial gradient in any pitch (Figure 10e).

## 4. Discussion

This study shows that mechanical surface properties measured using field-based instruments can differentiate polo pitches associated with different handicap levels, while also revealing within-pitch spatial variability that is not captured by summary statistics alone.

Results from the Linear Discriminant Analysis indicate that impact deceleration (ITD) was the primary variable contributing to separation among pitches across handicap levels. Separation along the first discriminant axis (LD1) was mainly associated with ITD, suggesting that impact response is an important mechanical characteristic distinguishing surfaces used at different levels of play. Other mechanical variables, including penetration resistance (GSP), shear resistance (GSS), and rotational peak shear (RPS), contributed less to between-pitch separation and appeared to describe variability within pitches rather than overall differentiation by handicap level. These findings are consistent with previous studies indicating that ITDC and related impact-based metrics are sensitive to differences in surface firmness and functional performance on equestrian sport surfaces [42,43,44].

The second discriminant axis (LD2) primarily reflected within-pitch variability and was most strongly associated with volumetric moisture content (VMC). This suggests that, particularly for pitches associated with higher handicap levels, differences in surface behavior are influenced not only by central tendency of mechanical properties but also by spatial consistency, especially in relation to moisture distribution. Variations in surface moisture content can influence the magnitude and distribution of loads exerted through the hoof during movement [37,52,53,54], thereby affecting multiple mechanical surface responses rather than a single functional parameter.

Spatial analysis further indicated that several mechanical parameters exhibited non-random spatial structure within pitches, expressed as directional gradients or localized patterns. Pitch A showed a pronounced moisture gradient, whereas Pitch B exhibited weaker and more localized spatial patterns, and Pitch C displayed comparatively uniform moisture distribution. These results indicate that surface conditions may vary substantially within individual pitches, even when median values fall within acceptable ranges. Although site-specific factors such as topography and soil heterogeneity may influence these patterns, causal attribution was not the objective of this study.

From an animal welfare perspective, controlled variability in surface loading may contribute to musculoskeletal adaptation during early stages of training, where exposure to variable and sub-maximal loads has been shown to support tissue conditioning [55]. However, when injury mechanisms are dominated by fatigue-related processes, such as stress fracture development [56,57,58], surface consistency becomes an important factor in reducing cumulative overloading. This consideration is particularly relevant in equestrian sports such as polo and horse racing, which involve high forces and grip demands, where localized surface inconsistencies may increase fatigue-related injury risk [18].

From a practical standpoint, these results highlight the limitations of relying solely on mean values or global indices to assess surface condition. Uniformity is a key functional property of equestrian surfaces, and spatial heterogeneity—particularly in impact response and moisture content—may influence both performance and safety by creating uneven footing conditions. While lightweight field-based tools do not replicate full equine biomechanical loading, they provide a practical means of identifying variability and supporting routine monitoring of surface condition.

Finally, the persistence of pitch differentiation by handicap level observed in this study suggests that the classification previously applied to these polo fields remains reflected in their current mechanical surface properties. Despite ongoing use and maintenance, the separation identified through multivariate analysis indicates that pitches continue to exhibit characteristic functional profiles aligned with their assigned handicap categories.

## 5. Conclusions

This study demonstrates that field-based mechanical measurements provide objective information on both global surface characteristics and within-pitch spatial variability of turf polo pitches. The combined use of univariate, multivariate, and spatial analyses enables differentiation of pitches associated with different handicap levels while also identifying internal heterogeneity that may not be apparent from qualitative assessment alone.

The existence of a handicap-based classification of polo pitches may facilitate alignment between surface characteristics and the functional demands of play. In higher-handicap competition, where game speed and spatial coverage are greater [6], surface properties related to impact response and consistency appear broadly compatible with these demands. However, the extent to which such alignment influences performance and equine welfare under competitive conditions remains to be fully evaluated.

At present, the interpretation of surface–performance relationships in polo is limited by the lack of comprehensive epidemiological datasets and longitudinal surface condition records comparable to those available for horse racing tracks. This constraint precludes direct associations between mechanical surface properties and injury risk, which are relatively well established in Thoroughbred racing [32,59]. Future research integrating surface monitoring with injury surveillance and performance metrics will be essential to establish evidence-based thresholds for surface management and to support welfare-oriented decision-making in polo.

## Figures and Tables

**Figure A1:**
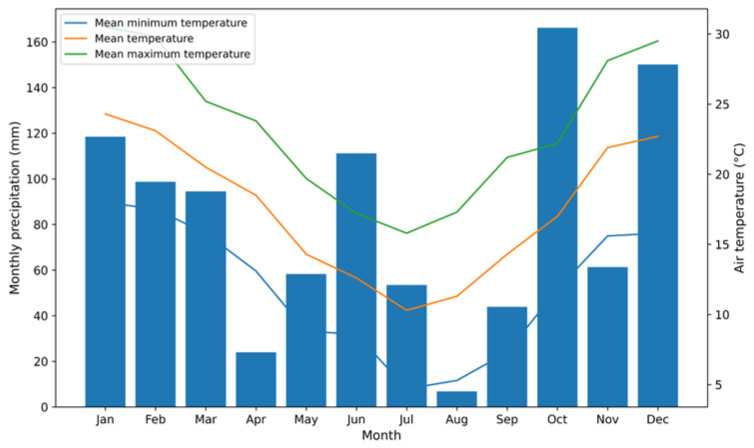
Monthly precipitation (bars, mm) and monthly mean air temperatures (lines: mean minimum, mean, and mean maximum; °C) recorded at the Mercedes Experimental Station during 2019. Precipitation is shown on the left *y*-axis and air temperature on the right *y*-axis [60].

**Figure A2:**
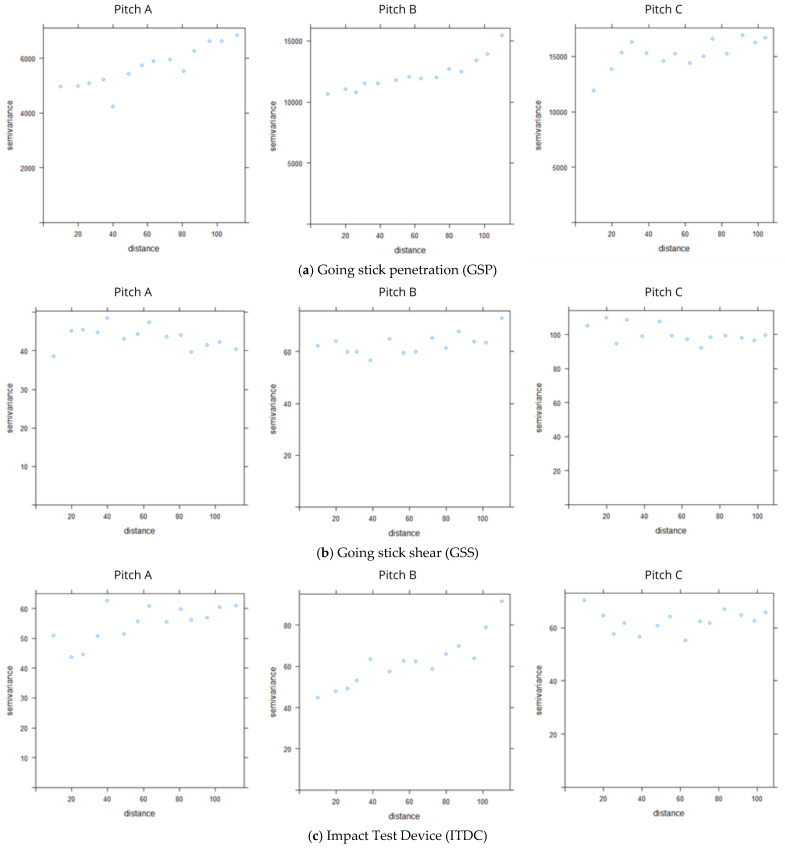

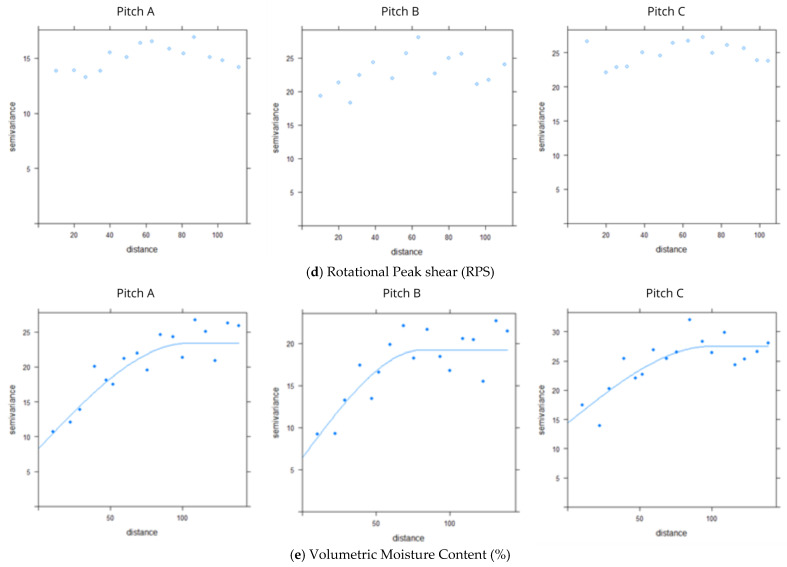
Sampled variograms constructed using the classical method-of-moments estimator proposed by Matheron and fitted models. Variograms are presented for all response variables across the three handicapped polo pitch types (A, B, and C). (**a**) Going Stick Penetration (GSP); (**b**) Going Stick Shear (GSS); (**c**) Impact Test Device Coefficient (ITDC); (**d**) Rotational Peak Shear (RPS); (**e**) Volumetric Moisture Content (VMC %). Spherical variogram models (solid line) were fitted only for VMC (%), as spatial dependence was consistently observed across all pitches for this variable. The other variables (GSP, GSS, ITD, RPS) were considered spatially independent (pure nugget effect).

## Data Availability

Data are available from the corresponding author upon reasonable request.

## Abbreviations

The following abbreviations are used in this manuscript:

ITD	Impact Test Device [G]
ITDC	Impact Test Device Coefficient [G]
GSS	Shear from Going Stick [Nm]
GSP	Penetration from Going Stick [N]
OBST	Orono Biomechanical Surface Tester
RPS	Rotational Peak Shear [Nm]
VMC %	Volumetric Moisture Content
FO	First Order
TWI	Two-way-interaction
PQ	Quadratic Term

## Appendix A

**Table A1:** Soil parameters obtained from samples of the three polo pitches surveyed.

Handicap Level	pH	C.E. (dS/m)	C.O. (%)	M.O. (%)	N (%)	C/N	P Bray 1 (ppm)	Ca (meq/100 g)	Mg (meq/100 g)
High	6.9	0.6	1.17	2.02	0.101	11.6	33.0	2.70	0.70
Medium	7.0	0.6	1.22	2.10	0.108	11.5	22.4	3.77	1.02
Low	6.6	0.5	1.51	2.60	0.114	13.2	10.1	11.88	3.04
Handicap level	K (meq/100 g)	Na (meq/100 g)	% Ca	% Mg	% K	CEC (meq/100 g)	ESP (%)	S-SO_4_ (ppm)	
High	0.30	0.39	73	18.9	8.1	3.7	10.5	8.3	
Medium	0.44	0.42	69.8	18.9	8.2	5.4	7.8	7.2	
Low	1.43	0.54	61.1	15.7	7.4	19.4	2.8	10.9	

Bray 1: P expressed in ppm; CEC (meq/100 g): calculated as the sum of exchangeable bases; C.E.: electrical conductivity of the saturated paste extract, (dS m^−1^); ESP: exchangeable sodium percentage.

## Appendix B. Spatial Autocorrelation Analysis of Response Variables

As a complementary step to the spatial interpolation presented in the main text, a preliminary assessment of spatial autocorrelation was conducted for each mechanical surface variable across all three polo fields. Prior to this analysis, large-scale trends were removed from the datasets to isolate local spatial structures. Following trend removal, spatial autocorrelation was only evident for the volumetric moisture content (VMC%), while the remaining response variables (GSP, GSS, ITD, RPS) could be considered spatially independent under the conditions of this study. However, this does not preclude the presence of directional trends or localized changes in surface behavior within the sampled area of each field.

Experimental variograms were constructed using the classical method-of-moments estimator proposed by Matheron and are presented below for each response variable and pitch type. Permissible variogram models were fitted only for VMC (%), where spatial dependence was consistently observed across all fields. These results justified the inclusion of spatial interpolation surfaces for VMC (%) in the main analysis and support the assumption of spatial independence for the other variables. For further guidance on variogram interpretation and spatial independence, see Ref. [50]).

## Appendix C. Surface Regression Models of Response Variables

**Table A2:** Sum of square partition and analysis of variance for the three main variation sources (FO: first-order, TWY: Two-way interaction, and PQ: Quadratic term) from the fitted surface regression models across the three pitch types (A, B, and C) and response variables: (**a**) Going Stick Penetration (GSP); (**b**) Going Stick Shear (GSS); (**c**) Impact Test Device (ITD); (**d**) Rotational Peak Shear (RPS); (**e**) Volumetric Moisture Content (VMC %). *p*-values lesser than 5% are indicated in bold.

(**a**) Going Stick Penetration Resistance					
A-HI	Df	Sum Sq	Mean Sq	F value	Pr(>F)
FO (x,y)	2.00	55,250.91	27,625.46	4.64	**0.01**
TWI (xy)	1.00	10,484.31	10,484.31	1.76	0.19
PQ (x^2^,y^2^)	2.00	2.20 × 10^5^	1.10 × 10^5^	18.45	**4.05 × 10^−8^**
Residuals	214.00	1.27 × 10 ^6^	5951.79		
B-MID	Df	Sum Sq	Mean Sq	F value	Pr(>F)
FO (x, y)	2.00	5.22 × 10^5^	2.61× 10^5^	22.27	**1.84 × 10^−9^**
TWI (xy)	1.00	74.46	74.46	6.36 × 10^−3^	0.94
PQ (x^2^, y^2^)	2.00	9.21 × 10^5^	4.61 × 10 ^5^	39.31	**3.91 × 10^−15^**
Residuals	201.00	2.35 × 10^6^	11,716.04		
C-LO	Df	Sum Sq	Mean Sq	F value	Pr(>F)
FO (x,y)	2.00	1.47 × 10^5^	73,506.15	4.63	**0.01**
TWI (x y)	1.00	34,034.78	34,034.78	2.14	0.14
PQ (x^2^, y^2^)	2.00	3.51 × 10 ^5^	1.76 × 10^5^	11.06	**2.77 × 10^−5^**
Residuals	201.00	3.19× 10 ^6^	15,882.39		
(**b**) Going Stick Longitudinal Shear					
A-HI	Df	Sum Sq	Mean Sq	F value	Pr(>F)
FO (x,y)	2.00	302.18	151.09	3.27	**0.04**
TWI (xy)	1.00	181.20	181.20	3.93	**0.05**
PQ (x^2^,y^2^)	2.00	90.82	45.41	0.98	0.38
Residuals	214.00	9,875.74	46.15		
B-MID	Df	Sum Sq	Mean Sq	F value	Pr(>F)
FO (x, y)	2.00	94.59	47.29	0.69	0.50
TWI (xy)	1.00	147.93	147.93	2.17	0.14
PQ (x^2^, y^2^)	2.00	168.78	84.39	1.24	0.29
Residuals	201.00	13,726.53	68.29		
C-LO	Df	Sum Sq	Mean Sq	F value	Pr(>F)
FO (x, y)	2.00	849.56	424.78	4.05	**0.02**
TWI (xy)	1.00	10.84	10.84	0.10	0.75
PQ (x^2^, y^2^)	2.00	299.48	149.74	1.43	0.24
Residuals	201.00	21,069.16	104.82		
(**c**) Impact Test Device—ITD					
A-HI	Df	Sum Sq	Mean Sq	F value	Pr(>F)
FO (x,y)	2.00	87.30	43.65	0.79	0.45
TWI (xy)	1.00	33.97	33.97	0.62	0.43
PQ (x^2^, y^2^)	2.00	747.14	373.57	6.79	**1.38 × 10 ^−3^**
Residuals	214.00	11,775.96	55.03		
B-MID	Df	Sum Sq	Mean Sq	F value	Pr(>F)
FO (x, y)	2.00	6207.95	3103.98	51.67	**7.84 × 10 ^−19^**
TWI (xy)	1.00	80.79	80.79	1.34	0.25
PQ (x^2^, y^2^)	2.00	5845.16	2922.58	48.65	**5.87 × 10 ^−18^**
Residuals	201.00	12,075.12	60.08		
C-LO	Df	Sum Sq	Mean Sq	F value	Pr(>F)
FO (x, y)	2.00	10,81.03	540.51	8.12	**4.08 × 10 ^−4^**
TWI (xy)	1.00	111.71	111.71	1.68	0.2
PQ (x^2^, y^2^)	2.00	730.66	365.33	5.49	**4.79 × 10 ^−3^**
Residuals	201.00	13,387.04	66.60		
(**d**) Rotational Peak Shear—RPS					
A-HI	Df	Sum Sq	Mean Sq	F value	Pr(>F)
FO (x,y)	2.00	547.51	273.75	20.54	**6.90 × 10^−9^**
TWI (xy)	1.00	0.11	0.11	8.53 × 10^−3^	0.93
PQ (x^2^, y^2^)	2.00	360.36	180.18	13.52	**2.95 × 10^−6^**
Residuals	214.00	2851.71	13.33		
B-MID	Df	Sum Sq	Mean Sq	F value	Pr(>F)
FO (x, y)	2.00	3426.40	1713.20	80.07	**2.66 × 10^−26^**
TWI (xy)	1.00	12.10	12.10	0.57	0.45
PQ (x^2^, y^2^)	2.00	283.57	141.78	6.63	**1.63 × 10^−3^**
Residuals	201.00	4,300.72	21.40		
C-LO	Df	Sum Sq	Mean Sq	F value	Pr(>F)
FO (x, y)	2.00	1014.90	507.45	20.26	**9.65 × 10^−9^**
TWI (xy)	1.00	45.09	45.09	1.80	0.18
PQ (x^2^, y^2^)	2.00	181.42	90.71	3.62	**0.03**
Residuals	201.00	5034.49	25.05		
(**e**) Volumetric Moisture Content					
A-HI	Df	Sum Sq	Mean Sq	F value	Pr(>F)
FO (x, y)	2.00	2978.07	1489.03	113.57	**2.42 × 10^−34^**
TWI (xy)	1.00	79.99	79.99	6.10	**0.01**
PQ (x^2^, y^2^)	2.00	2244.36	1122.18	85.59	**4.87 × 10^−28^**
Residuals	214.00	2805.67	13.11		
B-MID	Df	Sum Sq	Mean Sq	F value	Pr(>F)
FO (x, y)	2.00	534.31	267.16	18.27	**5.10 × 10^−8^**
TWI (xy)	1.00	65.27	65.27	4.46	**0.04**
PQ (x^2^, y^2^)	2.00	944.45	472.22	32.30	**6.85 × 10^−13^**
Residuals	201.00	2938.39	14.62		
C-LO	Df	Sum Sq	Mean Sq	F value	Pr(>F)
FO (x, y)	2.00	113.71	56.85	2.50	0.08
TWI (xy)	1.00	11.19	11.19	0.49	0.48
PQ (x^2^, y^2^)	2.00	698.07	349.04	15.33	**6.34 × 10^−7^**
Residuals	201.00	4575.09	22.76		
